# A comparison of NBI and WLI cystoscopy in detecting non-muscle-invasive bladder cancer: A prospective, randomized and multi-center study

**DOI:** 10.1038/srep10905

**Published:** 2015-06-05

**Authors:** Zhangqun Ye, Jia Hu, Xiaodong Song, Fan Li, Xuetao Zhao, Shan Chen, Xiaofeng Wang, Dalin He, Jinhai Fan, Dingwei Ye, Jinchun Xing, Tiejun Pan, Dongwen Wang

**Affiliations:** 1 Department of Urology, Tongji Hospital, Tongji Medical College, Huazhong University of Science and Technology, Wuhan 430030, China; 2 Department of Urology, Fourth Hospital of Wuhan, Wuhan 430030, P.R. China; 3 Department of Urology, Tongren Hospital, Peking 100730, P.R. China; 4 Department of Urology, People’s Hospital, Peking University, Peking 100044, P.R. China; 5 Department of Urology, First Affiliated Hospital, Xi’an Jiaotong University, Xi’an 710061, P.R. China; 6 Department of Urology, Cancer Hospital, Fudan University, Shanghai 200032, P.R. China; 7 Department of Urology, First Affiliated Hospital, Xiamen University, Xiamen 361003, P.R. China; 8 Department of Urology, Wuhan General Hospital of Guangzhou Military Region, Wuhan 430000, P.R. China; 9 Department of Urology, First Affiliated Hospital, Shanxi Medical University, Shanxi 030001, P.R. China

## Abstract

Several single-center studies have investigated whether narrow-band imaging (NBI) cystoscopy is more effective in detecting primary and recurrent non-muscle invasive bladder cancer (NMIBC) compared with white-light imaging (WLI) cystoscopy. In this study, we further evaluated the diagnostic value of NBI cystoscopy compared with WLI cystoscopy for primary NMIBC in a multi-center study. Suspected bladder cancer patients from 8 research centers received both NBI and WLI. Two experienced doctors in each center were responsible for the NBI and WLI assessments, respectively. The number of tumors and position of each tumor were recorded, and suspicious tissues were clamped and histologically examined. The sensitivity, specificity, and false-positive rate of NBI and WLI were evaluated. Of the 384 patients, 78 had a confirmed urothelial carcinoma (UC). The sensitivities of NBI and WLI were 97.70%, and 66.67%, respectively (*P* < 0.0001); the specificities were 50% and 25%, respectively; and the false positive rates were 50% and 75%, respectively. Based on 300 valid biopsy specimens, the NBI and WLI sensitivities were 98.80% and 75.45%, respectively (*P* < 0.0001). These results suggest that NBI has a high sensitivity and has superior early bladder tumor and carcinoma *in situ* (CIS) detection rates compared with WLI cystoscopy.

Bladder cancer is one of the most common cancers of the urinary system and has a high rate of recurrence^1^. Non-muscle invasive bladder cancer accounts for approximately 70%–80% of all bladder cancers^2^,^3^,^4^. Transurethral resection (TUR) with white-light imaging cystoscopy is the gold standard for detecting bladder neoplasms and monitoring patients for recurrence. However, WLI may fail to detect small papillary and subtle flat carcinoma *in situ* lesions, which could progress and become invasive^5^,^6^. More effective endoscopic methods are needed to detect bladder tumors due to these carcinomas. Using narrow band imaging technology, white light can be filtered into two narrow bands (415 nm and 540 nm), which are absorbed by hemoglobin, thereby increasing the visibility of the capillaries in the surface mucosa and the blood vessels in the submucosa^7^,^8^. Therefore, we hypothesize that NBI could enhance the contrast of superficial tumors to normal mucosa, which would improve the accuracy of bladder cancer diagnosis^9^,^10^. Recent studies from two institutions suggest that NBI cystoscopy improves the detection rate for primary and recurrent non-muscle invasive bladder cancer (NMIBC) compared with that of WLI^11^,^12^. We aimed to determine in a multi-center study whether NBI cystoscopy is more effective than WLI cystoscopy in the detection of primary NMIBC.

## Methods

### Study population and procedures

This study was approved by the ethics committee at each of the institutions (include Huazhong University of Science and Technology, capital medical university, Peking University, Xi’an Jiaotong University, Fudan University, Xiamen University, Wuhan General Hospital of Guangzhou Military Region and Shanxi Medical University). Written informed consent was obtained from each patient prior to blood sample collection. The methods were conducted in accordance with the approved guidelines. A total of 384 patients with a preliminary diagnosis of superficial bladder cancer were enrolled in this study at eight research centers; 267 patients were male, 117 were female, and the age ranged from 18 to 75. Each patient underwent WLI cystoscopy and NBI cystoscopy inspections in a randomized sequence conducted by two experienced urologists at each hospital. The procedures were randomly assigned to the urologists, who were unaware of the findings of their colleagues. The cystoscope used in our study was a flexible video cystoscope (Model CYF-VA2, Olympus Medical Systems, Tokyo, Japan) that easily be switched from the WLI model to the NBI model by pressing one button. After the endoscope was successfully placed into the patient’s bladder, each urologist had 10 minutes to complete the inspection and map the suspected areas under WLI or NBI on a standard bladder chart. After the procedures were completed, the biopsies were conducted by the urologists according to the corresponding chart. Under WLI or NBI cystoscopy, a biopsy of presumably normal urothelium would be performed at the end of the procedure (4 biopsies in the case of CIS). Biopsies in the new findings were not allowed. The specimens from different locations were separately placed in tagged containers. If the lesions detected using both methods were in the same location and were too small to obtain two biopsy samples, we performed one biopsy and placed the specimens in specifically tagged containers. The histopathology analysis was conducted by an institutional pathologist who was unaware of the detection method for each specimen. The ability of WLI and NBI cystoscopy to distinguish tumors from normal mucosa was compared according to the results of the biopsies.

### Power calculation

The WLI cystoscopy diagnostic rate is 93%–94%, as specified in the EAU/AUA guidelines. We set the margin of error at 5 percentage points so that the δ was 0.05 in our experiment; α = 0.05 was considered in our test. We used the following formula: N = (μα/δ)^2*p*^(1-p). In our test, μα = μ_0.05_ = 1.96; δ = 0.05; P = 0.93; n = (1.96/0.05)^2^ × 0.93 × (1 − 0.93) = 100. Hence, 103 subjects comprised an appropriate sample size.

### Outcome measures and statistics

The primary outcome of this study is a comparison of the sensitivity difference after NBI cystoscopy vs WLI cystoscopy in patients with NMIBC or CIS. As secondary outcomes, we compared the differences in the diagnostic specificity, YOUDEN index, positive likelihood ratio, negative likelihood ratio, positive predictive value, negative predictive value, kappa value and coincidence rate of both techniques in the patients with UC (primary or recurrent UC). The data were analyzed using SAS, version 8.1 (SAS Institute Inc., Cary, NC, USA), software and the EpiData Database. All the tests were two-tailed, with α = 0.05 as the type I error boundary.

## Results

**Figure 1:**
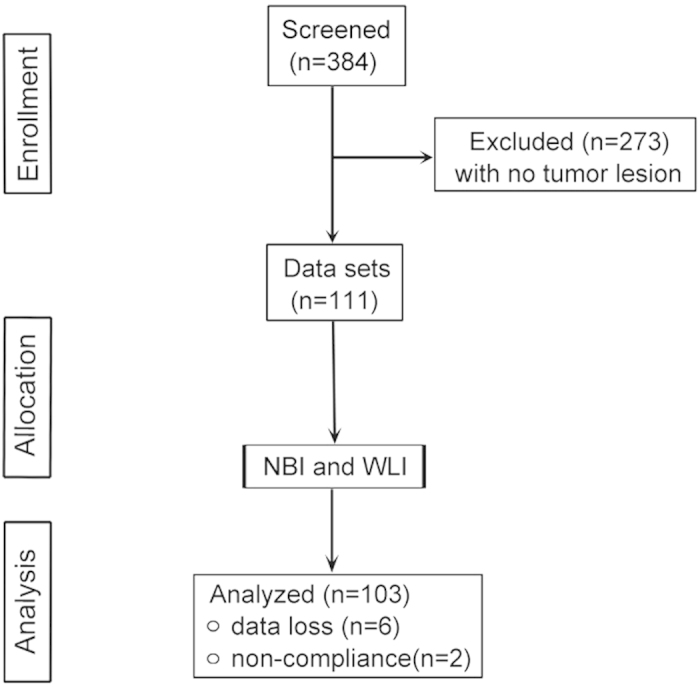


The demographic features found in the study are detailed in Table 1. A total of 273 patients with no tumor lesions were eliminated, and 8 patients were excluded because of data loss or non-compliance. The statistical analysis was conducted with the data from 103 patients (Fig. 1). The samples were divided into positive and negative groups based on the biopsy results. The patients with one or more positive samples were defined as positive (167 samples belonging to 87 patients). According to the WLI cystoscopy results, the number of positive patients and positive samples were 101 and 181, respectively. Similarly, the number of positive patients and positive samples were 103 and 213, respectively, based on the NBI cystoscopy results (Table 2).

A total of 167 specimens from 87 patients were confirmed as bladder cancer. A total of 41 samples from 29 of these patients were detected using NBI only, whereas 2 samples from 2 patients were detected using WLI only. Regarding the pathological stage, among the 41 NBI-detected samples, there were 16 samples in the pTa stage, 21 samples in the pT1 stage and 4 samples in the pTis stage; in comparison, the 2 WLI-detected samples were staged as pTa. No pTis stage sample was detected using WLI. The biopsy results of the positive groups are shown in Table 3. In addition, it is worth noting that above-mentioned 4 samples from 3 of patients in the pTis stage detected using NBI, whose preoperative urine cytology were all positive. This data indicates that NBI cystoscopy could complement urine cytology in diagnosis of CIS.

**Figure 2:**
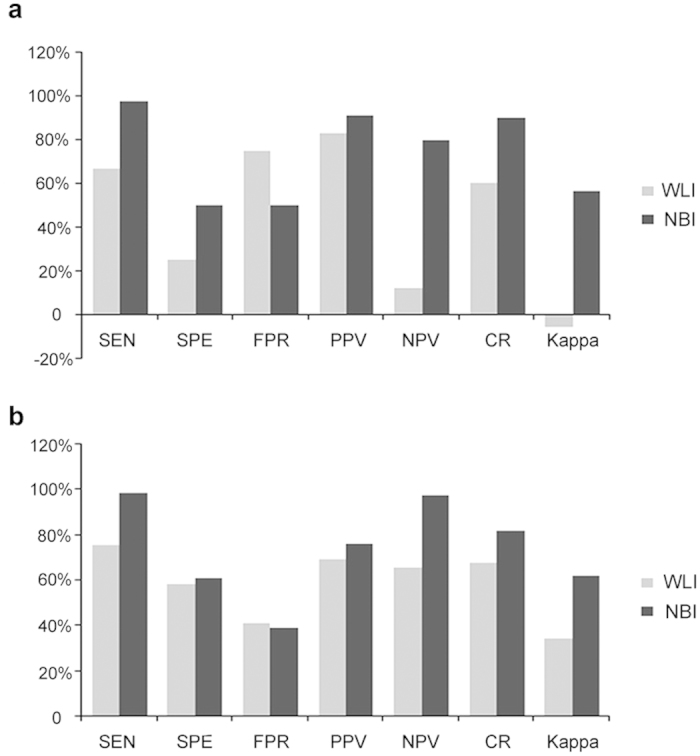
The comparison of statistical parameters between WLI and NBI (**A**) based on 103 patients (**B**) based on 300 biopsy specimens. SEN: sensitivity. SPE: specificity. FPR: false positive rate. PPV: positive predictive value. NPV: negative predictive value. CR: coincidence rate. Kappa: kappa value (consistency test).

To evaluate the diagnostic efficiency of conventional WLI cystoscopy and NBI cystoscopy, we calculated the specificity and the false positive rate (Fig. 2). With respect to 103 patients, the sensitivities of NBI and WLI were 97.70% and 66.67%, respectively (χ^2^ = 28.6140, *P* < 0.0001; the specificities of NBI and WLI were 50.00% and 25.00%, respectively (χ^2^ = 2.1333. *P* = 0.1441); the false positive rates of NBI and WLI were 50.00% and 75.00%, respectively (*P* = 0.1441); the positive predictive values of NBI and WLI were 91.40% and 82.86%, respectively (χ^2^ = 2.7064, *P* = 0.0999); and the negative predictive values of NBI and WLI were 80.00% and 12.12%, respectively (χ^2^ = 17.5755, *P* < 0.0001). In accordance with these evaluation indexes, the sensitivity, specificity, false positive rate, positive predictive value, and negative predictive value of NBI compared with those of WLI were 98.80% and 75.45% (χ^2^ = 40.5989, *P* < 0.0001); 60.90% and 58.65% (*P* = 0.7076); 39.10% and 41.35% (χ^2^ = 0.1407, *P* = 0.7076); 76.04% and 69.61% of WLI (χ^2^ = 2.0716, *P* = 0.1501); and 97.59% and 65.55% of WLI (χ^2^ = 29.9646, *P* < 0.0001), respectively, in 300 biopsy specimens.

## Discussion

Bladder cancer is one of the most common cancers in the urinary system and has a high rate of recurrence. Non-muscle invasive bladder cancer accounts for approximately 75%–85% of all bladder cancers^1^,^4^. Approximately 10%–20% of NMIBC progresses and develops into muscle invasive cancer^13^,^14^. However, in the early stages, carcinomas of the urinary bladder are invisible and very difficult to detect under conventional white light cystoscopy. The recurrence and progression of tumors are closely related to whether the tumor has been completely excised. Therefore, the early detection of bladder cancer is extremely important. Many new technologies have been developed to diagnose bladder cancer, including narrow band imaging, fluorescent cystoscopy, and chromoendoscopy^8^,^15^,^16^.

Narrow band imaging is a novel real-time optical image enhancement technology that uses a standard xenon light source in combination with unique narrow bandwidth color filters at a 415 ± 30 nm wavelength. The technology can be used to further narrow the bandwidth of the output light from the xenon light source to 514 ± 30 nm^8^,^9^. This narrow band of light penetrates only the surface of tissue and is absorbed by hemoglobin, thereby increasing the visibility of capillaries and other delicate tissue surface structures via contrast enhancement. NBI was first applied in the diagnosis of digestion system diseases and could be used to identify lesions in the esophagus, gastrointestinal tract and colon as well as differentiate tumors and precancerous lesions from normal tissue.

Recent reports have indicated that NBI cystoscopy is more effective than standard WLI cystoscopy for the detection of bladder cancer recurrence. In Herr’s study^10^,^17^, 90 of 103 (87.4%) recurrent bladder cancer patients were diagnosed using both WLI and NBI, whereas 13 (12.6%) were detected using only NBI cystoscopy. The mean number of recurrent tumors visualized under WLI cystoscopy was 2.3, whereas that of NBI cystoscopy was 3.4 (p = 0.01). Based on a small number of samples, Bryan^9^ reported that NBI was more effective than WLI in urothelial cancer diagnosis.

Our study was the first to evaluate the value of NBI for the detection of NMIBC in a multi-institutional setting in China. Compared with previous studies, a distinct feature of this research was that the WLI and NBI cystoscopy inspections in each center were conducted by two urologists who were experienced in cystoscopy. The order of the WLI or NBI inspections was randomized. The suspicious areas under WLI and NBI were first mapped on different charts and then biopsied according to each corresponding chart. The time consumed in each inspection was controlled to equivalence. The benefit of this design is the avoidance of mutual interference between the two exams. The same patients underwent examination to reduce errors resulting from differing research objectives. According to our clinical experience, NBI cystoscopy could be used to confirm a non-muscle-invasive bladder cancer diagnosis as well as determine the boundaries of the lesion. Particularly, *in situ* early stage tumors or carcinomas could be more clearly observed under NBI cystoscopy. According to our study, NBI improved the sensitivity of the diagnosis. Although the specificity and false positive rates of NBI cystoscopy(on the patient and specimen levels)were apparently superior to WLI cystoscopy, the statistical analysis revealed that there was no significant difference.

Although our study showed that NBI cystoscopy has high sensitivity and could improve the early detection of primary NMIBC over that of WLI, a few limitations of this study merit consideration. A number of doctors performed the WLI and NBI cystoscopy examinations; thus, the accuracy of NBI and WLI were subject to individual differences in the physician’s ability to detect tumors as well as to other subjective factors. Although the number of subjects of the study was lower than expected, future research studies could enroll more patients. There is no widely recognized standard for NBI diagnosis^18^, and the relationship between images and pathology should be more clearly defined. Additionally, a diagnostic standard for NBI images is necessary before NBI can be widely used in clinical diagnosis.NBI could be used to detect more tumors; however, whether NBI produces a meaningful endpoint is unknown. Anyhow, an accurate endoscopic exploration of the bladder may translate in less recurrences which could end in a more relaxed follow up and in less surgical endoscopic procedures as well, thus impacting significantly quality of life.

## Additional Information

**How to cite this article**: Ye, Z. *et al.* A comparison of NBI and WLI cystoscopy in detecting non-muscle-invasive bladder cancer: A prospective, randomized and multi-center study. *Sci. Rep.* **5**, 10905; doi: 10.1038/srep10905 (2015).

## Figures and Tables

**Table 1:** Demographic feature.

**Variable**	**Data**
**Gender**	
Male, n (%)	87(78.38)
Female, n (%)	24(21.62)
N (Missing)	111(0)
**Pregnant**	
Yes, n (%)	0 (0.00)
No, n (%)	24 (100.00)
Unknown, n (%)	0 (0.00)
**Age (yr)**	
Mean (SD)	61(10.7)
Median	62
Min~ Max	21 ~ 79
N (missing)	105(6)
**Smoking status**	
Yes, n (%)	32(28.83)
No, n (%)	79(71.17)
**Height (cm)**	
Mean (SD)	168.46(5.64)
Median	169.00
Min~ Max	152.00 ~ 180.00
N (missing)	106(5)
**Weight (kg)**	
Mean (SD)	67.05(9.36)
Median	67.5
Min	
Max 44	44.00 ~ 90.00
N (missing)	104(7)
**preoperative urine cytology findings**	
positive, n (%)	42(37.8)
negative, n (%)	57(51.3)
N (missing)	99(12)
**Primary or recurrent tumor**	
primary, n (%)	78(70.3)
recurrent, n	33(29.7)
**Prior bladder instillation therapy**	
Pirarubicin (THP), n	10
Hydroxycamptothecine (HCTP), n	2
Sapylin, n	1
**No. of suspicious tumors**	
Single, n (%)	54 (52.4)
Multiple, n (%)	49 (47.6)
**Tumor size**	
Small, n (%)	<1–2 cm 13 (12.6)
Medium, n (%)	2–5 cm 74 (71.8)
Large, n (%)	>5 cm 16 (15.6)
**Pathological findings of tumors**	
pT0, n (%)	16 (15.5)
pTa, n (%)	39 (37.9)
pTis, n (%)	11 (10.7)
pT1, n (%)	37 (35.9)

**Table 2:** The diagnostic results of WLI and NBI (N,%).

**Object**	**WLI**		**NBI**	
	**Positive**	**Negative**	**Positive**	**Negative**
Patients (n)	101(98.06%)	2(1.94%)	103(100.00%)	0(0.00%)
Samples (n)	181(60.33%)	119(39.67%)	213(71.00%)	87(29.00%)

**Table 3:** Concordance between each cystoscopic finding and the transurethral biopsy results.

**Results**	**Sample Level**	**Patient Level**
WLI + NBI−	2 (1.20% )	2 (2.30% )
WLI + NBI+	124 (74.25%)	56 (64.37%)
WLI − NBI+	41 (24.55%)	29 (33.33%)
Sum	167 (100.00%)	87 (100.00%)
